# Thyroid Hormones Interaction With Immune Response, Inflammation and Non-thyroidal Illness Syndrome

**DOI:** 10.3389/fcell.2020.614030

**Published:** 2021-01-21

**Authors:** Roberto De Luca, Paul J. Davis, Hung-Yun Lin, Fabio Gionfra, Zulema A. Percario, Elisabetta Affabris, Jens Z. Pedersen, Cinzia Marchese, Pankaj Trivedi, Eleni Anastasiadou, Roberto Negro, Sandra Incerpi

**Affiliations:** ^1^Department of Neurology, Center for Life Science, Beth Israel Deaconess Medical Center, Harvard Medical School, Boston, MA, United States; ^2^Pharmaceutical Research Institute, Albany College of Pharmacy and Health Sciences, Albany, NY, United States; ^3^Albany Medical College, Albany, NY, United States; ^4^Taipei Cancer Center, Taipei Medical University, Taipei, Taiwan; ^5^Graduate Institute of Cancer Biology and Drug Discovery, College of Medical Science and Technology, Taipei Medical University, Taipei, Taiwan; ^6^Traditional Herbal Medicine Research Center of Taipei Medical University Hospital, Taipei Medical University, Taipei, Taiwan; ^7^TMU Research Center of Cancer Translational Medicine, Taipei Medical University, Taipei, Taiwan; ^8^Department of Sciences, University “Roma Tre,” Rome, Italy; ^9^Department of Biology, University of Rome Tor Vergata, Rome, Italy; ^10^Department of Experimental Medicine, University “La Sapienza,” Rome, Italy; ^11^National Institute of Gastroenterology, Istituto di Ricovero e Cura a Carattere Scientifico (IRCCS) “S. de Bellis” Research Hospital, Castellana Grotte, Italy

**Keywords:** human immunodeficiency virus, hypothalamic–pituitary–thyroid, immune system, inflammasome, microRNAs, non-thyroidal illness syndrome, thyroid hormones, Wnt/β-catenin

## Abstract

The interdependence between thyroid hormones (THs), namely, thyroxine and triiodothyronine, and immune system is nowadays well-recognized, although not yet fully explored. Synthesis, conversion to a bioactive form, and release of THs in the circulation are events tightly supervised by the hypothalamic–pituitary–thyroid (HPT) axis. Newly synthesized THs induce leukocyte proliferation, migration, release of cytokines, and antibody production, triggering an immune response against either sterile or microbial insults. However, chronic patho-physiological alterations of the immune system, such as infection and inflammation, affect HPT axis and, as a direct consequence, THs mechanism of action. Herein, we revise the bidirectional crosstalk between THs and immune cells, required for the proper immune system feedback response among diverse circumstances. Available circulating THs do traffic in two distinct ways depending on the metabolic condition. Mechanistically, internalized THs form a stable complex with their specific receptors, which, upon direct or indirect binding to DNA, triggers a genomic response by activating transcriptional factors, such as those belonging to the Wnt/β-catenin pathway. Alternatively, THs engage integrin αvβ3 receptor on cell membrane and trigger a non-genomic response, which can also signal to the nucleus. In addition, we highlight THs-dependent inflammasome complex modulation and describe new crucial pathways involved in microRNA regulation by THs, in physiological and patho-physiological conditions, which modify the HPT axis and THs performances. Finally, we focus on the non-thyroidal illness syndrome in which the HPT axis is altered and, in turn, affects circulating levels of active THs as reported in viral infections, particularly in immunocompromised patients infected with human immunodeficiency virus.

## Introduction

Thyrotropin-releasing hormone (TRH) and thyroid-stimulating hormone (TSH) produced by the hypothalamus and pituitary gland, respectively, are effectors of the hypothalamic–pituitary–thyroid (HPT) axis, which regulates levels of circulating thyroid hormones (THs; Kelly, 2000). TRH induces TSH release that, once in circulation, stimulates THs biosynthesis and maturation, events that take place in the thyroid. The bioactive form of THs, namely 3,5,3′-triiodo-L-thyronine (T3; Incerpi et al., 2016), in turn, acts via a negative feedback loop to control the hypothalamic–pituitary component of the HPT axis (Kelly, 2000). T_3_ results from deiodination of thyroxine (T_4_) by deiodinase (DIO) 1 and 2 enzymes, while DIO 3 activity converts T_4_ in reverse T_3_ (rT_3_), an inert isomer of T_3_ (Incerpi et al., 2016; Lanni et al., 2016). T_3_ and T_4_ may enter into the target cells through specific transporters (Hennemann et al., 2001) and act by binding to different molecules located either on plasma membrane (i.e., integrin αvβ3; Bergh et al., 2005; Davis et al., 2005; De Vito et al., 2011) or intracellularly (i.e., THα and THβ receptors: THRs; Cheng et al., 2010; Brent, 2012; Incerpi et al., 2016). These interactions activate a variety of pathways that largely signal to the nucleus (Flamant et al., 2017), or the nuclear transcription machinery by directly activating THs response elements (TREs) on gene promoters (Singh et al., 2018). T_3_ shows higher affinity than T_4_ for THRs, whereas T_4_ is more potent than T_3_ in binding integrin avβ3. Both these receptors activate signaling molecules such as phosphoinositide 3-phosphate kinase (PI3K), protein kinase B (AKT), and mitogen-activated protein kinases (MAPKs; Incerpi et al., 2016; Lanni et al., 2016; Davis et al., 2019).

The immune system can also affect THs synthesis and release, either centrally (from thyroid gland), or peripherally, from tissues or target organs. Here, we review recent findings on how THs and the immune system crosstalk. In particular, we will focus on the THs-dependent regulation of (1) Nod-like receptor protein 3 (NLRP3)-mediated inflammasome, (2) small non-coding RNAs such as microRNAs (miRNAs; Anastasiadou et al., 2018a,b), and (3) Wnt/β-catenin pathway in anti- or pro-inflammatory conditions, such as non-thyroidal illness syndrome (NTIS) and (4) during chronic viral infections, such as those caused by human immunodeficiency virus (HIV).

## THs and Immune System: A Bidirectional Crosstalk

The existence of a bidirectional crosstalk between the endocrine and the immune system, in which THs and cytokines represent the key players, is well documented (Klecha et al., 2000, 2008; De Vito et al., 2011). Interestingly, immune cells’ reactivity to circulating THs (De Vito et al., 2011, 2012) as well as responsiveness of endocrine cells to available cytokines, such as interleukin-1 (IL-1), IL-6, interferon (IFN)-γ, and tumor necrosis factor-α (TFN-α), positively correlate with the expression of these molecules and to the affinity for their specific receptors (Klecha et al., 2000, 2008). A central role of THs in the modulation of immune system is confirmed by the influence of T_3_ and T_4_ in cytokine maturation and release, a process that involves the activation of MAPKs and mediated by phosphorylation of the Signal Transducer and Activator of Transcription 1α (STAT1α; Lin et al., 1999; Shih et al., 2004).

Abnormal THs secretion, hyperthyroidism, autoimmune thyroiditis, and hypothyroidism can affect immunological functions. Hyperthyroidism correlates with increased humoral and immune cell responses (De Vito et al., 2011). Opposite effects were found in hypothyroidism (Klecha et al., 2008). Moreover, levels of circulating THs positively match up with an immunological reactivity in healthy individuals, such as in physiological maintenance of lymphocyte subpopulations (Hodkinson et al., 2009). Recently, it has been shown that T_3_ increased the number of IL-17-expressing T lymphocytes by activating dendritic cells, *in vitro* (Alamino et al., 2019). In addition, T and B lymphocytes are capable of synthesizing and releasing TSH (Smith et al., 1983; Harbour et al., 1989), which might affect healthy and abnormal thyroid cells, expressing the TSH receptor. This novel and unexpected non-pituitary source of TSH could be also decisive in affecting immune response during infections and chronic inflammation (Klein, 2006). Initial reports of TSH and immune cells appeared more than 20 years ago (Smith et al., 1983; Kruger and Blalock, 1986). Bacterial toxins (Smith et al., 1983) or *in vitro* TRH administration (Klein, 2006) enhance TSH production and release from leukocytes. The work of Blalock et al. (1984) showed that TSH induced a strong cellular and humoral response, thus enhancing the lymphocyte proliferation by inducing the production of endogenous inflammatory factors: IL-6 and monocyte chemoattractant protein-1 (MCP-1; Gagnon et al., 2014). Moreover, *in vitro* and *in vivo* studies showed that TSH treatments significantly increased T_3_ levels in thymocytes and other immune cells (Csaba and Pállinger, 2009). Experiments performed in mice lacking the pituitary gland (unable to produce central TSH) showed increased THs levels during inflammation (Bagriacik et al., 2001). Conversely, unbalanced immune response may be linked to low levels of THs in the plasma, since TSH fluctuations might alter T_3_ and T_4_ release from thyroid gland. Moreover, acute infections indirectly influence THs release through the action of inflammatory molecules (like IL-1, IL-6, and TFN-α) on hypothalamus, thus minimizing TSH action on the thyroid and, consequently, reducing T_3_ and T_4_ in the circulation, promoting NTIS. This lowers the energy expenditure during illnesses, offering an alternative pathway to the HPT axis control, for central neuroendocrine–immune and metabolic fine-tuning (Klein, 2006). However, induction and regulation of NTIS may involve alterations in the HPT axis and may be relatively independent of circulating THs (de Vries et al., 2015).

**FIGURE 1:**
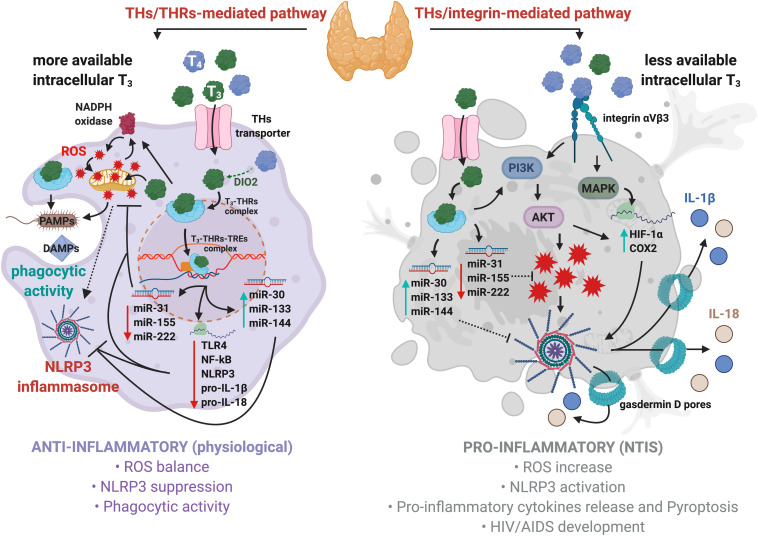
Regulation of NLRP3 inflammasome by thyroid hormones in macrophages. Physiological levels of T_3_ promote anti-inflammatory responses, bactericidal activity, and phagocytosis (left panel). Mechanistically, T_3_–THRs–TREs complex downregulates TLR4, NF-kB, NLRP3, pro-IL-1β, pro-IL-18, and several miRNAs, such as miR-31, -155, and -222, thus reducing ROS levels. Moreover, the T_3_–THRs–TREs complex upregulates miR-30, -133, and -144 that target Fasl, Ilk, Serpine1, hepatocyte growth factor (Hgf), Beta secretase 1 (Bace 1), and C-X-C motif chemokine receptor 4 (Cxcr4), thus further preventing the assembly of NLRP3 inflammasome (Forini et al., 2019). Hypothyroidism induces acute and chronic inflammatory responses, such as NTIS (right panel). High levels of T_4_ cause a robust production of ROS through the integrin αvβ3–PI3K–AKT signaling cascade, which ultimately triggers NLRP3 inflammasome. This is due to higher affinity displayed by T_4_ then T_3_ for integrin αvβ3 receptor on cell membrane. In addition, the T_4_–integrin αvβ3–MAPKs axis enhances the expression of HIF-1α and COX2 to promote NLRP3 inflammasome assembly and stability.

The T_3_ and T_4_ are also involved in the regulation of reactive oxygen species (ROS) production through the activation of the PI3K–AKT axis in immune cells (De Vito et al., 2011; Figure 1, right panel). Moderate levels of ROS could act as a second messenger and play an important role in the leukocyte activation during immune surveillance and phlogosis (Figure 1, left panel). This process, together with actin polymerization induced by T_4_ and rT_3_, may contribute to the immune cell migration and proliferation at the sites of inflammation (Marino et al., 2006; De Vito et al., 2011, 2012).

## Role of THs on NLRP3 Inflammasome Activation

Inflammasomes are intracellular multiprotein complexes typical of immune cells, such as monocytes and macrophages, which mediate the first line of defense in response to sterile (absence of microbial particles) and non-sterile (microbial infection) threats, by activating pro-inflammatory cytokines (He et al., 2016a,b; Mangan et al., 2018). The sterile signals include damage-associated molecular patterns (DAMPs; Ahechu et al., 2018), debris from dead or dying cells (Newton and Dixit, 2012), and other organic and inorganic molecules (Allam et al., 2013; He et al., 2016a,b; Amores-Iniesta et al., 2017). The non-sterile agents encompass the pathogen-associated molecular patterns (PAMPs), lipopolysaccharide (LPS; Schroder and Tschopp, 2010), RNA (Franchi et al., 2014), and a wide range of bacterial toxins (Greaney et al., 2015).

Inflammasomes consist of a sensor protein, such as NLRP3, which recognizes the insults and activates effector proteins: Caspase-1. The active Caspase-1 cleaves the inflammatory pro-IL-1β and pro-IL-18 to generate their mature forms, as well as gasdermin D, whose N-terminus domains auto-assemble into pores on the plasma membrane for the release of bioactive cytokines, thus inducing an inflammatory form of cell death known as pyroptosis (Shi et al., 2015; Magupalli et al., 2020). Two temporally distinct events are required for the full activation of NLRP3-mediated inflammasome. The first step, *priming*, involves engagement of Toll-like receptors (TLRs) by pathogens or sterile particles. This is followed by recruitment of the myddosome complex, which transduce downstream signal to NF-kB, allowing an increase of NLRP3 and pro-ILs levels (Lamkanfi, 2011). The second event, *activation*, consists in the assembly of the inflammasome proteins into a functional active structure and includes different signal molecules, which cause an intracellular ion disbalance and activation of ROS production, culminating with NLRP3 inflammasome maturation (Wang L. et al., 2020). The amplitude of the inflammasome activation is a crucial event that controls shifts from acute to severe inflammation (Moossavi et al., 2018; Wang Z. et al., 2020). Recent evidence suggests that negative or positive modulation of the NLRP3 inflammasome could be dependent on T_3_ availability and uptake in the target cells, thus possibly diverting a physiological condition toward a pathological status. Therefore, T_3_ activity could be crucial for adequate macrophage function and tissue homeostasis. Indeed, alterations in these processes could lead to cancer, diabetes, intestinal bowel disease, or atherosclerosis (Wynn et al., 2013; Kwakkel et al., 2014).

After uptake, T_3_ partially migrates to the nucleus and binds to the macrophage dominant isoform of THRs (i.e., THRα; Kwakkel et al., 2014) and, subsequently, to the TREs located on promoters of the target genes. The T_3_–THRs–TREs complex regulates gene transcription through direct or indirect interactions with the nuclear DNA (Singh et al., 2017). It is well-established that the T_3_–THRs–TREs complex affects different miRNAs families, as miR-30, -133, and -144, whose expressions are increased by T_3_–THRs–TREs complex activity (Forini et al., 2018, 2019). These miRNAs dampen pro-inflammatory genes, such as Fast apoptosis signal Ligand (FasL; Chen et al., 2016) and Integrin-linked kinase (Ilk), two key players that trigger NLRP3 inflammasome assembly and inflammation (Boro and Balaji, 2017). Moreover, it was recently shown that the T_3_–THRs–TREs complex reduced cardiac-related miR-31, -155, and -222 (Forini et al., 2018). This results in an increased expression of superoxide dismutase 1 (SOD1) and 2 (SOD2; Wang et al., 2015; Forini et al., 2019), which lower the levels of ROS and inhibit the activation of NLRP3 inflammasome. In addition, the T_3_–THRs–TREs complex downregulates the TLR4/NF-kB pathway (Furuya et al., 2017; de Castro et al., 2018), thus reducing the levels of NLRP3, pro-IL-1β, and pro-IL-18. All this suggests that T_3_–THRs nuclear action may direct immune cells to an anti-inflammatory condition (Vargas and Videla, 2017; Forini et al., 2019; Figure 1, left panel). In particular, cytosolic T_3_–THRs complex controls nicotinamide adenine dinucleotide phosphate oxidase (NADPH)-dependent ROS production by involving the PI3K–AKT axis (Gnocchi et al., 2012). Cytosolic ROS partially contribute to the generation of mitochondrial ROS (mtROS; West et al., 2011; Pushpakumar et al., 2017), thus forming a loop between NADPH and mitochondria, which keeps intracellular levels of ROS within a physiological range (Dikalov, 2011). Finally, the cooperative interactions between the T_3_–THRs complex, moderate levels of ROS and mtROS, maintain the NLRP3 inflammasome activation under strict control and promote bactericidal clearance, phagocytic activity, and anti-inflammatory condition (Vernon and Tang, 2013; van der Spek et al., 2018; Figure 1, left panel).

On the other hand, more pronounced pro-inflammatory pathways might take place when levels of THs lean toward T_4_, a common condition diagnosed in clinical hypothyroidism, often associated with inflammation and risk of NTIS onset (Boelen et al., 2004; Mancini et al., 2016). Circulating T_4_ binds to integrin αvβ3, located on plasma membrane and signals to MAPKs, thus increasing levels of hypoxia-inducible factor 1-alpha (HIF-1α) and cyclooxygenase-2 (COX2; De Vito et al., 2011; Lin H. Y. et al., 2013a), both involved in the NLRP3 inflammasome activation (Hua et al., 2015; Gupta et al., 2017). In parallel, the T_4_–integrin αvβ3 axis activates PI3K and AKT, thus inducing a robust production of ROS (De Vito et al., 2012), as well as enhancing HIF-1α expression (Lin H. Y. et al., 2013a; Hsieh et al., 2017). All these events could ultimately lead to NLRP3 inflammasome activation (Figure 1, right panel). In support of this, it was found that excessive iodine promoted pyroptosis of thyroid follicular cells by the ROS–NF–kB–NLRP3 pathway in a model of autoimmune thyroiditis (Liu et al., 2019).

In summary, the net immunological response is determined by concentration and availability of circulating and intracellular THs, as well as by the metabolic status that could potentially promote an anti- or pro-inflammatory response by opposite regulations on NLRP3 inflammasome activation and stability.

## Interplay Between THs, Wnt Pathway, and miRNAs During Inflammation

**FIGURE 2:**
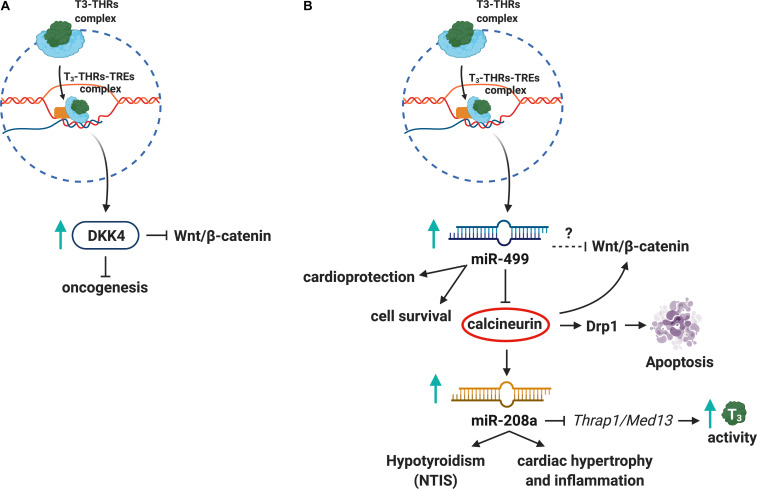
Thyroid hormones, Wnt pathway, and miRNAs crosstalk. **(A)** The T_3_–THRs–TREs complex upregulates DKK4, a tumor suppressor, which inhibits Wnt/β-catenin pathway. **(B)** Also, the T_3_–THRs–TREs complex increases miR-499 and downregulates calcineurin and miR208a, thus reducing apoptosis, cardiac hypertrophy, inflammation, and NTIS.

Interactions between THs and the Wnt/β-catenin pathway have been investigated in recent years (Todaro et al., 2010). The modulation of Wnt/β-catenin signaling pathway by the T_3_–THRs–TREs complex affects fundamental biological processes such as cell proliferation, development, tissue homeostasis, and metabolism (Ely et al., 2018). While THRα1 receptor controls gut development and homeostasis through the Wnt pathway in physiological conditions (Kress et al., 2009), in pathological conditions, such as colorectal cancer, the THRα1 receptor is thought to activate β-catenin/Tcf4 transcription, thus increasing the cell proliferation and tissue rearrangement in the gut (Kress et al., 2010). Grainyhead-like transcription factor 3 (GRHL3), essential for epidermal differentiation and morphogenesis, suppresses DIO3 activity (increasing T3 levels) and acts as a downstream signal for the Wnt/β-catenin pathway (Kimura-Yoshida et al., 2018). In addition, the T_3_–THRs–TREs complex induces expression of Dickkopf (DKK) 4, which antagonizes Wnt/β-catenin signaling in hepatocellular carcinoma cell lines (HCC), suggesting a role for T_3_ in tumor suppression and unraveling the T_3_/DKK4/Wnt/β-catenin pathway as a possible therapeutic target in HCC (Liao et al., 2012; Figure 2A). The T_3_–THRs–TREs complex also controls several epigenetic mechanisms of gene expression (Dong et al., 2010; Janssen et al., 2014; Forini et al., 2018). Dissecting T_3_–THRs–TREs-dependent genetic and epigenetic crosstalk could provide new insights to develop therapeutic strategies for pathologies that affect the HPT axis, as NTIS and autoimmune thyroiditis (Tomer, 2014; McDermott, 2019*). Decreased levels of THRs and THs have been found in animal models for NTIS and in NTIS patients affected by sepsis and cardiovascular disturbances (Warner and Beckett, 2010; von Hafe et al., 2019).

Several studies showed that T_3_–THRs–TREs signaling affects miRNAs expression (Dong et al., 2010; Janssen et al., 2014; Babu and Tay, 2019). For instance, T_3_–THRs–TREs binds to miR-17 promoter and decreases transcription and processing of mature miR-17, involved in cancer (Lin Y. H. et al., 2013b). Also, some miRNAs regulated by THs play an important role in the Wnt pathway. For instance, miR-499 related to cardioprotection (Wang et al., 2011) inhibits calcineurin (a signaling molecule of Wnt/β-catenin pathway), thus reducing the levels of Dynamin-1-like protein (Drp1), which is involved in apoptosis (Tan et al., 2008). Another cardiac-specific miRNA (Seok et al., 2014; Wang et al., 2015; Su et al., 2016), miR-208a, inhibits T_3_-mediated signaling pathway by repressing the THs Associated Protein/Mediator Complex Subunit 13 (THRAP1/MED13) in a mouse model of cardiac hypertrophy and hypothyroidism (van Rooij et al., 2009; Neppl and Wang, 2014; Figure 2B). Although there is ample evidence about the THs/THRs/miRNAs alterations in immune-related pathologies, it will be crucial to further explore the regulatory networks between miRNAs and THs in pathological contexts such as NTIS.

## NTIS and THs During HIV Infection

Impairments in the HPT axis in the course of NTIS affect circulating levels of THs, especially T_3_. NTIS may result from HPT setpoint alterations that occur during prolonged hospitalization in a variety of systemic diseases (Boelen et al., 2011; de Vries et al., 2015; Yasar et al., 2015). In this context, it has been described that patients affected by severe acute respiratory syndrome (SARS) present signs of NTIS and HPT dysfunctions (Marazuela et al., 2020; Pal and Banerjee, 2020). Same alterations might also be caused by Coronavirus disease 2019 (COVID-19) as recently reported (Khoo et al., 2020; Wai Lui et al., 2020). However, other factors such as elevated levels of circulating IL-6 (Wajner et al., 2011) and TNF-α (Feelders et al., 1999) also inhibit or reduce T_4_ conversion to T_3_ in NTIS patients.

NTIS is characterized by reduced circulating levels of T_3_ and increased rT_3_, as a result of dysregulated deiodination of intracellular T_4_ by DIO3 and perhaps other deiodinases, which inactivate THs, preventing their excess (De Groot, 1999; Wajner et al., 2011). In particular, LPS administration is a model of NTIS that stimulates DIO2 activity, NF-κB activation, and consequently cytokine increase, whereas it decreases DIO1 levels (Boelen et al., 2011). Furthermore, many systemic and non-endocrine pathologies such as congestive heart failure, cardiorenal syndrome, and starvation/malnutrition are commonly observed in NTIS patients (Larsen et al., 2002; Lee and Farwell, 2016). It has been suggested that NTIS may represent a form of hypothyroidism linked to the oxidative stress and reduced antioxidant defense system, related to the altered function of deiodinases (Mancini et al., 2016). In fact, the administration of the antioxidant N-acetylcysteine, in order to prevent NTIS in patients with acute myocardial infarction, increased serum T_3_ while decreasing rT_3_ (Vidart et al., 2014; de Vries et al., 2015; Lee and Farwell, 2016). Interestingly, adaptive NTIS response, in terms of thyroid function, during sustained immune defense has been interpreted as an effort—in terms of reduced available T_3_—to decrease the energy expenditure and turnover of several proteins involved in host defenses (Klein, 2006; de Vries et al., 2015). Acute NTIS has also been interpreted as a support mechanism for the immune response because of high production of pro-inflammatory cytokines found at the early stage of the disease (Boelen et al., 2011).

Acquired immunodeficiency syndrome (AIDS), in which HIV seriously compromises immune defenses, is associated with dysfunction of HPT and endocrine organs and shows typical markers of endocrine alterations related to NTIS, such as high ROS levels (Parsa and Bhangoo, 2013). More importantly, recent findings suggest that HIV-related conditions promote NRLP3-mediated inflammasome activation (Haque et al., 2016; Bandera et al., 2018; Figure 1, right panel).

The screening of TSH is highly recommended in HIV patients and, if the levels of TSH are found altered, free T_3_ and T_4_ measurements become necessary. During such screenings, possible occurrence of NTIS must be considered for differential diagnosis related to the abnormal thyroid functionality, especially in individuals with advanced AIDS (Hoffmann and Brown, 2007). Analysis of THs metabolism on *post-mortem* tissues of HIV-infected patients showed alterations in the HPT axis and 27% of screened HIV patients showed abnormal TSH levels (50% had TSH < 0.5 mU/L and the remaining had >4 mU/L; Langford et al., 2011). HPT axis alterations are usually considered as NTIS and depend on the severity of HIV-related disease. TSH may change, and usually the activity of deiodinases is decreased; therefore, higher circulating T_4_ and rT_3_ levels are found, whereas circulating T_3_ is decreased (Hoffmann and Brown, 2007). The lack of effectiveness of T_3_ administration/replacement in NTIS patients (Chopra, 1997; De Groot, 2006; Warner and Beckett, 2010; de Vries et al., 2015) could be explained by the actions of rT_3_, which is largely inactive (Lanni et al., 1993, 2016; Moreno et al., 2008).

The autoantibodies, namely, TgAb and TPOAb, were also altered in HIV-infected individuals (Ketsamathi et al., 2006). Drug abuse, or its withdrawal, may also contribute to this (Langford et al., 2011). Interestingly, during anti-retroviral therapy (ART), a subclinical hypothyroidism is commonly observed. Indeed, isolated levels of TSH are elevated, whereas low free T_4_ is found (Hoffmann and Brown, 2007). Similarly, results on THs alterations have been reported in a study on HIV-infected children treated with ART. Therefore, due to the serious outcome of these pathologies and their consequences on the psychosomatic development, the thyroid dysfunctions should be carefully evaluated not only in adults but also in children (Viganò et al., 2004). Indeed, impact of NTIS in critically ill children (Jacobs et al., 2019) remains unclear.

## Discussion and Future Perspectives

Herein, we have discussed the bilateral crosstalk between the immune system and THs both in physiological and patho-physiological conditions. The activation of NLRP3 inflammasome, modulation of the Wnt/β-catenin pathway, and NTIS could rely on a complex interplay that involves THs and miRNAs. Finally, how viral infections could affect NTIS and HPT functions have been discussed. Broadly, we have provided new insights into how the immune system and endocrine system interact with each other. Ultimately, it is our hope that ideas discussed here will eventually open novel avenues of research and drug development (Silverman et al., 2020). In particular, since miRNAs are involved in the crosstalk between inflammation and THs-related diseases, they might be considered not only as biomarkers but also as potential druggable targets in order to combat, with higher efficiency, NTIS. Indeed, a recent study has shown how downregulation of miR-155 by an anti-miRNA compound, cobomarsen, reduced inflammation and tumor volume in preclinical models and in a patient (Anastasiadou et al., 2020). Future studies related to how THs affect the immune system in physiological and pathological settings, including those that mimic HIV infection *in vitro*, will provide important insights and impetus to this exciting field.

## Author Contributions

RDL, PJD, PT, EAn, RN, and SI conceptualized and wrote the manuscript. H-YL, FG, ZAP, EAf, JZP, and CM edited the manuscript and provided important insights and suggestions. All authors contributed to the article and approved the submitted version.

## Conflict of Interest

The authors declare that the research was conducted in the absence of any commercial or financial relationships that could be construed as a potential conflict of interest.
